# *Leishmania* infection and blood sources analysis in *Phlebotomus chinensis* (Diptera: Psychodidae) along extension region of the loess plateau, China

**DOI:** 10.1186/s40249-020-00746-8

**Published:** 2020-08-31

**Authors:** Han-Ming Chen, Hui-Ying Chen, Feng Tao, Jing-Peng Gao, Kai-Li Li, Hua Shi, Heng Peng, Ya-Jun Ma

**Affiliations:** 1 Department of Naval Medicine, Naval Medical University, 800 Xiangyin Road, Shanghai, 200433 China; 2 Institute of Disease Control and Prevention of People’s Liberation Army of China, Beijing, 100071 China; 3 Department of Medical Microbiology and Parasitology, College of Basic Medical Sciences, Naval Medical University, 800 Xiangyin Road, Shanghai, 200433 China

**Keywords:** Sand fly, *Leishmania*, Blood source, Extension region of the loess plateau, China

## Abstract

**Background:**

Visceral leishmaniasis (VL) was one of the most important parasitic diseases in China, caused by *Leishmania* protozoans and transmitted by sand flies. Recently VL cases have reappeared in China, including the extension region of the Loess Plateau. The purpose of this study was to collect fundamental data on the host-vector VL system in the Loess Plateau to assist in the development of prevention and control measures.

**Methods:**

Sand flies were collected by light traps from rural areas in Shanxian, Henan, China in 2015, as well as in Wuxiang and Yangquan, Shanxi, China in 2017. The blood sources of sand flies were analyzed by PCR detecting the host-specific mitochondrial cytochrome *b* (mtDNA *cyt b*) gene fragments. *Leishmania* infection in sand flies was detected by amplifying and sequencing ribosomal DNA internal transcribed spacer 1 (*ITS1*). The *Leishmania* specific antibodies in the sera of local dogs were detected by ELISA kit.

**Results:**

Blood sources showed diversity in the extension region of the Loess Plateau, including human, chicken, dog, cattle, pig and goat. Multiple blood sources within a sand fly were observed in samples from Yangquan (17/118, 14.4%) and Wuxiang (12/108, 11.1%). *Leishmania* DNA was detected in sand flies collected from Yangquan with minimum infection rate of 1.00%. The *ITS1* sequences were conserved with the *Leishmania donovani* complex. The positive rate of *Leishmania* specific antibodies in dogs was 5.97%.

**Conclusions:**

This study detected the blood sources and *Leishmania* parasites infection of sand flies by molecular methods in the extension region of Loess Plateau, China. A high epidemic risk of leishmaniasis is currently indicated by the results as the infection of *Leishmania* in sand flies, the extensive blood sources of sand flies including humans, and positive antibody of *Leishmania* in local dog sera. Given the recent increase of VL cases, asymptomatic patients, dogs and other potential infected animals should be screened and treated. Furthermore, the density of sand flies needs to be controlled and personal protection should be strengthened.

## Background

Visceral leishmaniasis (VL), also known as kala-azar, is a sand fly borne disease caused by *Leishmania* protozoans. VL was one of the most important parasitic diseases in China [1–4]. At present, VL is mainly endemic in western China, and cases occurred in Xinjiang, Inner Mongolia, Gansu, Sichuan, Shaanxi, and Shanxi [2, 3]. From 2004 to 2012, a total of 3337 cases were reported, of which 97.03% were distributed in Xinjiang, Gansu and Sichuan [5]. In 2018, 180 cases of VL were reported in 78 counties of 11 provinces in China, mainly distributed in Gansu, Shanxi and Shaanxi Province, and the endemic area has expanded [6], indicating the rising risk of VL in China.

Three endemic types of VL have been described in China, the anthroponotic type (AVL), the zoonotic desert type (DT-ZVL) and zoonotic mountain type (MT-ZVL) [7]. AVL was endemic in the Kashgar alluvial plain and the Aksu oasis of Xinjiang, and it was historically called “anthroponotic” because no reservoir animal was ever found. DT-ZVL is dominant in ancient oases and deserts in Xinjiang and Inner Mongolia, and although in some places it, too, has an unknown reservoir, the Tarim hare (*Lepus yarkandensis*) has been identified as its reservoir in some endemic areas. The extension region of Loess Plateau in Shanxi, Henan and Shaanxi Provinces was one of the typical MT-ZVL endemic regions. The characteristics of the MT-ZVL include: the pathogen was mainly *Leishmania infantum*, the vector was *Phlebotomus chinensis*, and the zoonotic hosts were dog (*Canis familiaris*) as well as raccoon dog (*Nyctereutes procyonoides*) [8–11].

In China, VL was eliminated in most endemic areas after stringent implementation of control programs by the government in the 1950s [8]. However, local VL cases began to reappear since the early twenty-first century [12, 13]. In 2018, 38 VL cases were reported in Shanxi Province, mainly in Yangquan City (15 cases) and Pingding County (8 cases). In addition, three and 27 cases were reported in Henan and Shaanxi provinces [6].

The reason of VL recurrence in the extension region of Loess Plateau was puzzled, which was an obstacle to develop prevention and control measures. Blood meal identification is important evidence for the determination of the host preferences of hematophagous arthropods. Detection of *Leishmania* infection in sand flies and animal hosts could provide critical information to estimate the vector competence and assess the epidemic risk of VL in the endemic areas [14, 15]. However, there was no report on *Leishmania* infection and blood source animals of sand flies in the extension region of Loess Plateau.

In order to explore the reasons for the VL recurrence in the area, we collected sand flies from three sites in the extension region of the Loess Plateau, China including Shanxian in Henan Province, Yangquan and Wuxiang in Shanxi Province. *Leishmania* infection and blood sources were detected, and the *Leishmania* specific antibodies were determined in the sera of local dogs.

## Materials and methods

### Ethics statement

This study was carried out in strict accordance with the National Natural Science Foundation of China ethical guidelines for biomedical research involving living animals and human subjects (2016).

### Sand fly collection and species identification

**Fig. 1:**
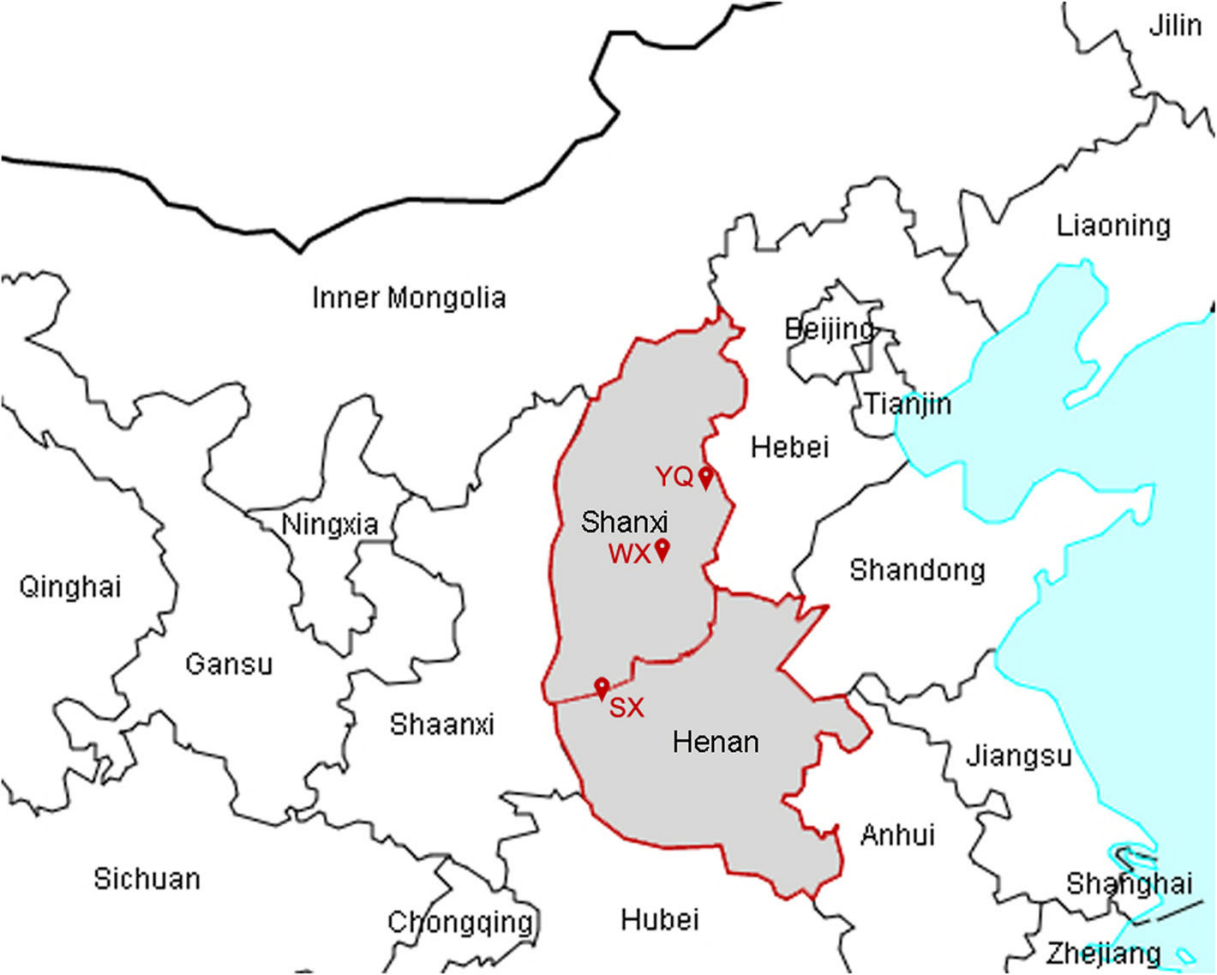
Schematic diagram of the collection location. SX, Xizhang Village, Shanxian County, Henan Province, China; YQ, Hedi Village, Yangquan City, Shanxi Province, China; WX, Moyu Village, Wuxiang County, Shanxi Province, China

The sand fly samples were collected at three sites located in the extension region of the Loess Plateau, China (Table 1, Fig. 1): Xizhang Village (111.22°E/34.62°N, 985 m) in Shanxian County (SX), Henan Province in July 2015, Hedi Village (113.56°E/38.00°N, 895 m) in Yangquan City (YQ) and Moyu Village (113.09°E/36.79°N, 1050 m) in Wuxiang County (WX), Shanxi Province, China in June 2017.

**Table 1:** The information on sample collection of sandflies in the extension of the Loess Plateau, China

Collection site		Date	Longitude	Latitude	Altitude (m)
Xizhang Village, Shanxian County, Henan Province, China (SX)	Chicken sheds, cave dwelling and courtyard	July 2015	111.22°E	34.62°N	985
Hedi Village, Yangquan City, Shanxi Province, China (YQ)	Chicken farm, utility room and courtyard	June 2017	113.56°E	38.00°N	895
Moyu Village, Wuxiang County, Shanxi Province, China (WX)	Livestock sheds	June 2017	113.09°E	36.79°N	1050

Light traps (MYFS-HJY-1, Houji Dianzi, Dongguan, China) were used to catch sand flies. With the consent of the owners, light traps were set up in utility room, cave dwelling, courtyard and chicken farm from 5:30 pm to 8:30 am, and collected manually in the evening by mouth aspirators. The captured sand flies were sorted and counted by male and female, respectively.

Some fresh female sand fly adults were randomly selected to dissect to check our molecular identifications. The species were identified in according with the morphology of pharyngeal armature and spermatheca [16]. The rest of the specimens were preserved in RNAfixer (Aidlab Biotechnologies, China) and brought back to the laboratory. All samples used in this study were identified by DNA sequences. Blood source analysis was conducted on those female specimens with visible blood residues, whereas all female sand flies were used to detect *Leishmania* infection.

### The ecological niches of the sand flies

The ecological niches of the sand flies in the extension region of the Loess Plateau China were described in our published article [17]. In brief, the collection sites are located in hilly lands with altitude ranges from 895 m to 1050 m, with similar geographical features and typical northern temperate climate. The buildings are cave dwellings or brick houses with tile roof. There are a variety of domesticated animals in the villages, including chickens, dogs, pigs, cattle and goats. Most animals were kept in caves or semi-closed livestock circles adjacent to the houses, and some animals are kept open in the courtyard.

### DNA extraction and molecular identification of sand flies species

Genomic DNA of sand fly samples was extracted using DNAzol (Life Technologies, USA) following the manufacturer’s instructions. The fragment of the mitochondrial cytochrome *b* (mtDNA *cyt b*) gene of sand flies was amplified according to the method reported by Esseghir [18]. The primers were forward CB1 (5′-TAT GTA CTA CCA TGA GGA CAA ATA TC-3′) and reverse CB3-R3A (5′-GCT AAT TAC TCC TCC TAA CTT ATT-3′). The positive PCR products were sequenced using four-color fluorescently labeled dideoxy termination method in Boshang Biotech Co., Ltd. (Shanghai, China). The sequences were Blast aligned in GenBank on the NCBI website (https://blast.ncbi.nlm.nih.gov/Blast.cgi) to determine the sand fly species.

### Blood sources identification

The female sand flies with visible blood residues were used for blood sources analysis, including 31 pooled and 226 individual samples. There were 12 pools from SX, 9 pools from YQ and 10 pools from WX. Every pooled sample contained 10 individuals. The individual samples include 118 from YQ and 108 from WX. The mtDNA *cyt b* fragments of different animals and human were amplified by PCR [19–22]. According to the main animal species in the collection site, PCR assay was developed with primers specific to human, chicken, goat, pig, cattle and dog. The information of primer sets was listed in Table 2, and separate PCRs were performed for each pair of primers. The PCR reaction was carried out in 25 μl containing 1.5 μl DNA template, 0.2 μmol/L primers and 12.5 μl 2 × PCR mix reagents (Aidlab Biotechnologies, China). The PCR running parameters were starting at 94 °C for 2 min; continuing with 35 cycles of 94 °C for 15 s, 51 °C for 30 s, and 72 °C for 1 min; and a final extension with 72 °C for 8 min. The PCR products were electrophoresed on a 1.5% agarose gel to determine the size and were sequenced to confirm.

**Table 2:** PCR primers for mtDNA-*cytb* amplification of blood sources of sandflies

Species	Primer	Sequence (5′ → 3′)	Amplicon length (bp)
Human (*Homo sapiens*)	HF	GGC TTA CTT CTC TTC ATT CTC TCC T	334
	UR	GGT TGT CCT CCA ATT CAT GTT A	
Chicken (*Gallus gallus*)	ChF	CAT ACT CCC TCA CTC CCC CA	802
	ChR	CCC CTC AGG CTC ACT CTA CT	
Goat (*Capra hircus*)	GoatF	AAT CAT CCG ATA CAT ACA CG	506
	GoatR	ATA TAG TTG TCT GGG TCT CC	
Pig (*Sus domesticus*)	PF	CCT CGC AGC CGT ACA TCT C	453
	UR	GGT TGT CCT CCA ATT CAT GTT A	
Cattle (*Bos taurus*)	CF	CAT CGG CAC AAA TTT AGT CG	561
	UR	GGT TGT CCT CCA ATT CAT GTT A	
Dog (*Canis familiaris*)	DF	GGA ATT GTA CTA TTA TTC GCA ACC AT	680
	UR	GGT TGT CCT CCA ATT CAT GTT A	

### *Leishmania* spp. detection in sand flies

*Leishmania* infection was identified in all female sand flies. The ribosomal DNA internal transcribed spacer 1 (*ITS 1*) fragment of *Leishmania* was amplified using the following primers: forward LITSR 5′-CTG GAT CAT TTT CCG ATG-3′, reverse L5.8S 5′-TGA TAC CAC TCG CAC TT-3′ [23]. The PCR reaction was performed in 25 μl contained 1.5 μl DNA template, 0.2 μmol/L primers and 12.5 μl 2 × PCR mix reagents. The PCR temperature profile was as follows: starting at 94 °C for 2 min; continuing with 35 cycles of 94 °C for 30 s, 52 °C for 30 s, and 72 °C for 30 s; and a final extension with 72 °C for 8 min. A positive control containing *Leishmania donovani* DNA (provided by National Institute of Parasitic Diseases, Chinese Center for Disease Control and Prevention) and a negative control without DNA template were utilized.

### Detection of anti-*Leishmania* antibody in the sera of dogs

The sera of 67 dogs (*Canis familiaris*) from YQ were provided by Yangquan Center for Disease Control and Prevention. The dogs were 0.5–14 years old. The owners of the dogs had been informed and consented to the blood collection. The sera were separated by centrifugation at 1000×*g* for 10 min after blood collection in the field. Then the sera were refrigerated and transported to the laboratory for testing. The *Leishmania* specific antibodies were detected using the commercialized Dogs *Leishmania* Ab ELISA kit (Fusheng Industrial Co., Ltd. Shanghai, China). A 96-well ELISA plate coated with *Leishmania* antigen, positive and negative control sera were provided in the kit. The serum samples were diluted and assayed according to the manufacturers’ instructions. Briefly, diluted serum samples, positive and negative control sera were added to the 96-well ELISA plate coated with antigen. After incubation and subsequent washing, the HRP-conjugate antigen was added to the wells. The plate was washed again after incubation. Then the HRP enzyme substrate was added for chromogenic reaction. The optical density (OD) value was recorded at 450 nm using a photometer (BioTek, Gene Company Ltd., USA). Each sample was tested in triplicate. The samples were considered positive if the mean OD value was higher than the positive cut-off value.

## Results

### Sand fly species identification

Thirty specimens were identified by morphology as *Ph. chinensis*. All other samples used in this study were identified as *Ph. chinensis* by mtDNA *cyt b* gene sequencing, which was conserved with the HM747267 sequence in GenBank database. The *Ph. chinensis* is the absolutely dominant sand fly species in the extension region of the Loess Plateau China.

### Blood sources identification

The blood sources of the fed sand flies was according to the mtDNA *cyt b* fragment of the animal species (Supplementary Fig. 1), include human (*Homo sapiens*, MT735217), pig (*Sus domesticus*, MT735219), chicken (*Gallus gallus*, MT735216), goat (*Capra hircus*, MT735218), dog (*Canis familiaris*, MT735215) and cattle (*Bos taurus*, MT735214). In SX, chicken blood was positive in all 12 pooled samples (100%), followed by cattle (66.7%), human (33.3%), dog (33.3%) and pig (16.7%), whereas goat was negative in all samples. In YQ, chicken and human were the most common blood sources. Chicken blood was identified in 88.9% of pooled samples and 59.3% of individuals, and human blood was positive in 66.7% pooled samples and 26.3% individuals. In WX, goat blood was found in all pooled samples (100%) and 34.3% individuals; human blood was detected in 70.0% pooled samples and 64.8% individuals. Chicken blood was positive in 10.0% pooled samples and 21.3% individuals. Dog blood was positive in 10.0% pooled samples and 0.9% individuals (Table 3). In general, the sand fly has a wide range of blood sources in the extension region of the Loess Plateau, China.

**Table 3:** Blood sources detection results of the fed sandflies in the extension region of the Loess Plateau, China

Collection site	Samples (*n*)	Blood sources, number of positive samples (%)					
		Human	Pig	Cattle	Dog	Chicken	Goat
SX	Pooled samples (12)	4 (33.3%)	2 (16.7%)	8 (66.7%)	4 (33.3%)	12 (100%)	0 (0.0%)
YQ	Pooled samples (9)	6 (66.7%)	0 (0.0%)	0 (0.0%)	0 (0.0%)	8 (88.9%)	0 (0.0%)
	Individual samples (118)	31 (26.3%)	0 (0.0%)	0 (0.0%)	0 (0.0%)	70 (59.3%)	1 (0.8%)
WX	Pooled samples (10)	7 (70.0%)	0 (0.0%)	0 (0.0%)	1 (10.0%)	1 (10.0%)	10 (100%)
	Individual samples (108)	70 (64.8%)	0 (0.0%)	0 (0.0%)	1 (0.9%)	23 (21.3%)	37 (34.3%)

SX, Shanxian; YQ, Yangquan; WX, Wuxiang

Multiple blood sources within a sand fly were found in 54 individuals. Among them, 17 individual samples collected from YQ (14.4%) with human plus chicken blood sources. The other 37 individual sand flies were collected from WX (34.3%), including chicken + human (*n* = 10), goat + human (*n* = 24), chicken + goat (*n* = 1), chicken + goat + human (*n* = 1) and dog + goat + human (*n* = 1).

### *Leishmania* infection in sand flies

*Leishmania ITS 1* product were positive in five pooled samples (5/59, 8.5%) and two individual specimens (2/108, 1.9%) from YQ population (Supplementary Fig. 2). The minimum infection rate of *Leishmania* in the sand fly population of YQ was 1.00% (7/698). All sequences of *Leishmania ITS 1* amplicons (MH200624) were conserved with *L. donovani* complex (*L. donovani*/*L. infantum*) [24]. Of the two positive individual specimens, one had human blood meal, and the other had chicken blood meal. No sample of SX and WX populations was positive in *Leishmania* DNA detection.

### Detection of *Leishmania* specific antibodies in dog sera

Out of the 67 dog sera samples, 4 were identified as positive (5.97%) in the detection of *Leishmania* specific antibodies according to the cut-off value using Dogs *Leishmania* Ab ELISA. The four positive dogs had no obvious pathological manifestations such as hair removal, desquamation, mental wilting, etc.

### Comprehensive detections from Yangquan

Comprehensive results were obtained in the samples collected from Yangquan, Shanxi Province, China, which were summarized as below: i) A total of 3599 sand flies were collected, and *Ph. chinensis* was absolutely dominant species. ii) Chicken and human were the most common blood sources, and multiple blood sources within a sand fly were observed. iii) *Leishmania* DNA was detected in five pooled samples and two individual specimens, which sequences were conserved with *L. donovani* complex (*L. donovani/L. infantum*). iv) The positive rate of *Leishmania* specific antibodies in dog sera was 5.97% (4/67).

## Discussion

Recent VL cases along the extension region of the Loess Plateau have been of the MT-ZVL type and have occurred mostly in children less than ten years old [2, 3]. The primary vector of MT-ZVL is *Ph. chinensis*, which feeds on various animals as well as humans. We have verified dogs as a reservoir host of *Leishmania*; it may be the principal reservoir and the key to human VL cases in the region. A high *Leishmania* infection rate has been detected in dogs in the past, and human VL cases have been reduced after the elimination and suppression of local infected dogs [11, 25, 26]. Recently, VL cases in extension region of the Loess Plateau, China reappear after the disease eradication for 20 years, which posed a challenge for control.

Many sand fly species are known to rely on a broad range of species for blood meals [19, 20, 27–29], typically mammals [20, 30–33] but also various cold-blooded animals [21]. In this study, we investigated the blood sources of sand flies by amplified specific animal’s mtDNA *cyt b* fragments using PCR. The results showed that the chickens and humans were the most common blood sources of sand flies in the extension region of the Loess Plateau, China, while dogs, goats, cattle and pigs were also blood sources. In Jiuzhaigou of Sichuan, China, swine was the dominant blood source of sand flies, followed by chickens and dogs [22]. The results do not support the generally accepted opinion that humans and dogs are preferred feeding host of *Ph. chinensis*. As a good and sufficient blood source in the region, chickens may contribute to the sustainability of a large sand fly population, which was similar to other investigations [34, 35]. Although chickens attract sand flies in the extension region of the Loess Plateau, China and in Brazil [35], the role of chickens in the epidemiology of the sand fly-borne diseases has not been defined yet. In this study, the proportions of blood sources were different among the three collection sites. The reason for this difference seems to be related to the blood sources available around the sandflies, depending on the environment.

We verified dogs as a reservoir host using samples from Yangquan, but in this region, unlike in Shanxian and Wuxiang, we did not detect dogs as a blood meal source of sand flies. However, Yangquan sand fly collection sites were far from areas frequented by dogs, and in the other regions, where the ecologic niches of sand flies were similar to that in Yangquan, dog was detected as a blood meal source. Thus we believe that dogs are likely a blood source for sand flies in Yangquan, and dogs are commonly seen in sand fly habitats there. Nonetheless, these data suggest caution in drawing final conclusions about the host-vector dynamics leading to VL infections in this region. Indeed, *Leishmania* spp. have been detected by PCR assay, such as in bovines (5%), buffaloes (4%), goats (16%) [36], and even desert lizards [37]. In fact, goats are believed to constitute a reservoir host of *L. donovani* in Nepal [7]. Therefore, other reservoir animal hosts of *Leishmania* should be further investigated. Consequently, we believed that dogs were still the blood source of sand flies in Yangquan, which frequently moving around sand flies’ habitats. In the next step, the sample size of the sandflies for blood sources analysis should be expanded. Multiple blood sources were found in individual fed specimens, suggesting a complex feeding behavior, which was critical for sand flies to transmit zoonotic diseases to humans.

In China, vector sand flies with *Leishmania* infection have been reported in Sichuan and Shaanxi provinces [16, 38]. The natural infection rate of a new haplotype of *L. donovani* was 1.98% in some villages in Sichuan Province [39], consistent with the results of sand fly infection rate (minimum infection rate 1.00%) in this study. To the best of our knowledge, this study was the first report of sand flies infected with *Leishmania* spp. in the extension region of the Loess Plateau, China. Monitoring natural *Leishmania* infection in sand flies provides critical information to assess the local epidemic risk of VL situation. Of the seven positive samples, the amplified sequences were all conserved with *L. donovani* complex (*L. donovani*/*L. infantum*). Multiple lines of evidence suggested that there were heterogeneous *Leishmania* strains in China. These strains were distinct from but phylogenetically related to *L. donovani*/*L. infantum* complex [24, 26, 37, 39, 40]. In this study, part of the *ITS1* fragment sequence was amplified for *Leishmania* detection, which was not sufficient for typing the *Leishmania*. It would be interesting to isolate the local Leishmania species and determine its type using more variable regions of the genome.

Dogs were confirmed as the reservoir host of *Leishmania* spp. in mountainous type of zoonotic VL in the extension region of Loess Plateau, China [2, 3]. It has been reported that the positive rate of *Leishmania* spp. in dogs was above 50% in Jiuzhaigou of Sichuan, 41.9% in Heishui of Sichuan, and 77.21% in Wenxian of Gansu, China [11, 25, 41]. In Shanxi Province, there was only one investigation in 1959, in which the *Leishmania* positive rate in dogs was 0.01% [16]. In this study, the antibodies positive rate in dog sera from Yangquan was 5.97%. Among the seven positive sand fly samples tested, at least three contained human blood. Based on the preceding investigation, we believed that the screening and treatment of the disease sources need to be strengthened including asymptomatic infected persons and dogs.

Although we have detected the blood sources, *Leishmania* infection and the antibody of *Leishmania* in dog sera, there were still some limitations in this study. First, the primers for PCR detection of blood sources were designed based on the observation of the environment of the collection sites, but some blood sources animals may be missed because they are not observed. Moreover, the pooled samples were used for blood sources and *Leishmania* infection detection, making the positive rates not accurate. In addition, the investigation of potential reservoir hosts was insufficient in the study. Besides the dogs, other animals may serve as reservoir host. And the best way to verify *Leishmania* infection was to visualize the parasite under a microscope or isolate the culture from the samples. In the future, we will collect more animal blood samples to detect *Leishmania* infection and antibodies, and try to isolate the parasite from the samples, so as to provide more accurate guidance to prevent and control of VL in the extension region of Loess Plateau in China.

## Conclusions

This study detected the blood sources and *Leishmania* parasites infection of sand flies by molecular methods in the extension region of Loess Plateau, China. The following comprehensive information indicates that the current risk of leishmaniasis endemic is high: high density of *Ph. chinensis* vector, the positive infection of *Leishmania* in sand flies, the diverse blood sources of sand flies including humans and dogs, and positive antibody of *Leishmania* in local dog sera. Given the recent increase of VL cases, asymptomatic patients, dogs and other potential infected animals should be screened and treated. Furthermore, the density of sand flies needs to be controlled and personal protection should be strengthened.

## Supplementary information

**Additional file 1: Supplementary Fig. 1** Agarose gel electrophoresis of PCR products for the detection of sandfly blood sources. A, chicken mtDNA *cyt b* gene amplification, lane 1, DNA marker; lane 2–14, detection samples. B, human mtDNA *cyt b* gene amplification, lane 1, DNA marker; lane 2–13, detection samples. C, goat mtDNA *cyt b* gene amplification, lane 1, DNA marker; lane 2–15, detection samples

**Additional file 2 Supplementary Fig. 2** Agarose gel electrophoresis of PCR products for *Leishmania ITS1* detection in sand flies. Lane 1, positive control; lane 3–12, detection samples; lane 13, negative control. Lane 7, 9 and 11 were positve samples

## Data Availability

The datasets used and/or analyzed during the current study are available from the corresponding author on reasonable request.

## Abbreviations

DNA: Deoxyribonucleic acid
VL: Visceral leishmaniasis
PCR: Polymerase Chain Reaction
ELISA: Enzyme Linked Immunosorbent Assay
*cyt b*: Mitochondrial cytochrome b
*ITS1*: Internal transcribed spacer 1
YQ: Yangquan
WX: Wuxiang
SX: Shanxian
