# Supplementary figures and images for: Leishmania infection and blood sources analysis in Phlebotomus chinensis (Diptera: Psychodidae) along extension region of the loess plateau, China
Source: Infect Dis Poverty. 2020 Aug 31;9:125. doi: 10.1186/s40249-020-00746-8 (PMC7461359; doi:10.1186/s40249-020-00746-8)

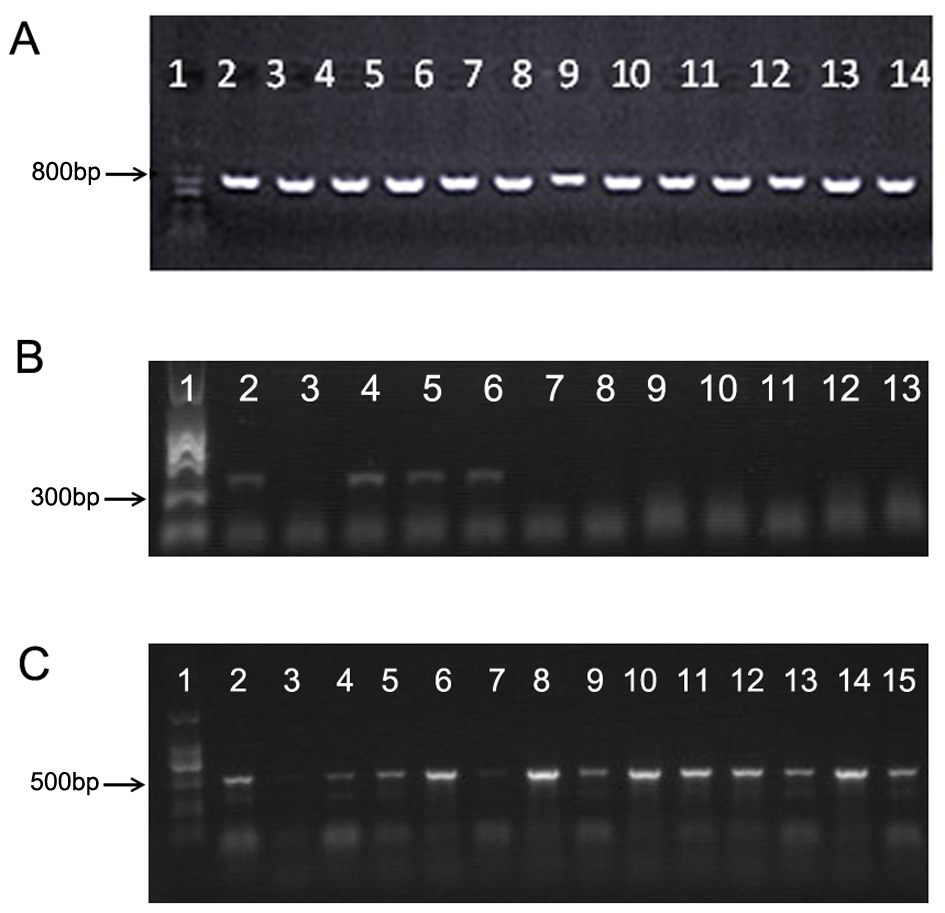

Supplement: Supplementary file 1 — Additional file 1: Supplementary Fig. 1 Agarose gel electrophoresis of PCR products for the detection of sandfly blood sources. A, chicken mtDNA cyt b gene amplification, lane 1, DNA marker; lane 2–14, detection samples. B, human mtDNA cyt b gene amplification, lane 1, DNA marker; lane 2–13, detection samples. C, goat mtDNA cyt b gene amplification, lane 1, DNA marker; lane 2–15, detection samples [file 40249_2020_746_MOESM1_ESM.jpg]

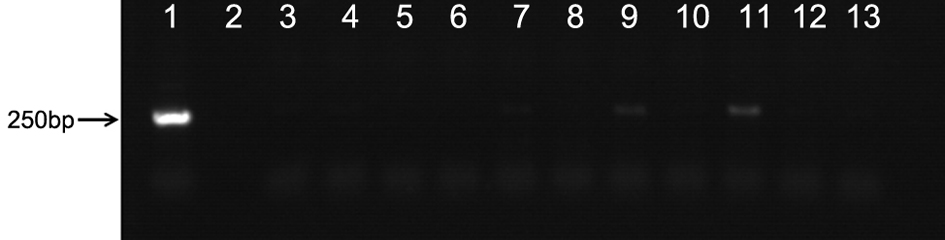

Supplement: Supplementary file 2 — Additional file 2 Supplementary Fig. 2 Agarose gel electrophoresis of PCR products for Leishmania ITS1 detection in sand flies. Lane 1, positive control; lane 3–12, detection samples; lane 13, negative control. Lane 7, 9 and 11 were positve samples [file 40249_2020_746_MOESM2_ESM.jpg]
